# Are specialized sperm function tests clinically useful in planning assisted reproductive technology?

**DOI:** 10.1590/S1677-5538.IBJU.2020.01.03

**Published:** 2020-01-13

**Authors:** Sandro C. Esteves

**Affiliations:** 1 ANDROFERT, Clínica de Andrologia e Reprodução Humana, Centro de Referência para Reprodução Masculina, Campinas, SP, Brasil; 2 Departamento de Cirurgia (Disciplina de Urologia), Universidade Estadual de Campinas (UNICAMP), Campinas, SP, Brasil; 3 Faculty of Health, Aarhus University, Aarhus, Denmark

**Keywords:** Infertility, Male, Therapeutics, Reproductive Techniques, Assisted

## Abstract

40-year-old male patient and 32-year-old female partner, with a history of primary infertility of two years duration. The workup revealed idiopathic mild oligoasthenotheratozoospermia, and no apparent female infertility factors. The couple has failed three intrauterine insemination (IUI) cycles, planning more IUI cycles but also considering in vitro fertilization/intracytoplasmic sperm injection (IVF/ICSI).

## INTRODUCTION

The use of sperm function tests in the above scenario is only valid if test results help us to guide clinical management and provide benefit to patients at acceptable costs. Reviewing the published literature over the last five years, specifically concerning the clinical utility of specialized sperm function tests (SSFTs) for male infertility diagnosis, intrauterine insemination (IUI), and assisted reproductive technology (ART) outcomes, it becomes evident that the current research shifted to tests that assess sperm function at the molecular level. Nevertheless, a few studies still explore the use of sperm fertilizing ability, such as sperm capacitation and acrosome reaction (AR), as potential biomarkers of sperm function.

### Tests evaluating sperm fertilizing ability

In a 2018 prospective study, Schinfeld and colleagues (1) evaluated pregnancy rates according to the results of a commercially available assay that measures the sperm fertilizing ability by assessing capacitation in 91 couples undergoing IUI. The authors found that the likelihood of achieving pregnancy by IUI was about 3-fold higher when sperm with the so-called ‘high fertilizing potential’ was used for insemination. According to this study, the sperm capacitation test could help the clinician to decide whether a couple should insist on IUI or better move to ART.

In another 2018 study, Xu and colleagues (2) examined the utility of the AR test to predict fertilization in 485 couples undergoing conventional in vitro fertilization (IVF). The authors found that the percentage of sperm exhibiting spontaneous AR in neat semen predicted fertilization rates (FR). An area under the curve (AUC) of 0.85 (p=0.005) for low (FR<30%) or total fertilization failure was achieved using a threshold of 9.5%. Thus, according to their results, the AR test could help the doctor to decide whether a couple is eligible for conventional IVF or better move to intracytoplasmic sperm injection (ICSI).

### Tests measuring oxidative stress

Oxidative stress (OS) tests have also been explored concerning male infertility diagnosis and ART outcomes. In a 2017 study by Agarwal and colleagues, the authors measured the oxidation-reduction potential (ORP) in 594 infertile men and 101 fertile controls using a commercially available assay, which provides a snapshot of the balance between oxidants and reductants in the semen. They found that that ORP levels were twice higher among infertile men (vs. controls; p<0.001), and that the ORP cutoff of 1.42 mV/10^6^ sperm distinguished fertile from infertile men with 60.6% sensitivity, 74.3% specificity, and 93.3% positive predictive value (3).

Using the same assay, a study from South Africa involving 51 couples showed that ORP results might be useful to predict low fertilization (defined as FR<66.7%) as well as live birth rates (LBR) by ICSI, with AUCs of 0.81 (p=0.002) and 0.77 (p=0.0007), respectively (4). The authors also showed that ORP results were correlated with sperm DNA fragmentation (SDF) values obtained with the use of the TUNEL assay (r=0.53; p=0.0001), which is not surprising given the well-known association between OS and SDF.

### Tests evaluating sperm DNA damage

Most current data on the clinical utility of SSFTs concern SDF tests. In fact, results of a 2017 survey study showed that SDF tests are commonly requested by fertility specialists, with TUNEL, sperm chromatin structure assay (SCSA), sperm chromatin dispersion (SCD) test, and Comet assay being the four most common assays utilized to measure SDF (5). The average reported cost for a SDF test was 170 US dollars (±123). Among the top 3 indications for test requests, i.e., (i) pregnancy failure after IUI, IVF, or ICSI, (ii) recurrent miscarriage after IUI, IVF, or ICSI, and (iii) recurrent natural pregnancy loss (RPL), the case of couples with failed IUI or ART ranked first. Interestingly, about two out of three responders said that abnormal SDF levels would affect their clinical management.

These results are actually not surprising given the well-established association between sperm DNA damage and risk of infertility (6-11). It has also been suggested that SDF negatively affects IUI and ART outcomes (12-15). Moreover, the sperm genetic defect could be transferred via ART, thus potentially affecting the health of resulting offspring (16, 17).

Indeed, big data from a compilation of 28 studies including 1294 fertile men and 2883 infertile men indicate that SDF testing can be a potent tool for male infertility diagnosis, with thresholds of 20% having high accuracy (AUC: 0.844; p<0.01) to distinguish fertile from subfertile men (18). These results are consistent with those of a landmark study by Ribas-Maynou et al., which showed that there exist high correlations (r>0.70; p<0.001) and similar thresholds among SCSA (18.9%), SCD (22.7%), and TUNEL (20.0%) values concerning male infertility diagnosis. In the above study, the alkaline Comet assay was also accurate (AUC: 0.93) for male infertility diagnosis, but with higher SDF thresholds (45.4%) than the three other tests (19).

In our Clinic, SDF results in a consecutive cohort of 1639 men - using the SCD test - indicate that over 50% of the patients have values of 20% and higher. And 25% of our population have SDF values of 30% or higher (20). In general, our group follows the practice recommendations issued by the Society for Translational Medicine in 2017 to request the SDF test (21). According to these evidence-based guidelines, the main clinical scenarios for testing include (i) Clinical varicocele (in particular, grades 2 and 3 varicocele with normal conventional semen parameters, and grade 1 varicocele with borderline/abnormal conventional semen parameters), (ii) Unexplained infertility/IUI failure/RPL, (iii) IVF and/or ICSI failure, and (iv) Borderline abnormal (or normal) semen parameters with risk factor (e.g., smoking, obesity, gonadotoxin exposure).

IUI failure seems to be an excellent indication to recommend the test as patients with high SDF would better benefit from ART than IUI. In a 2007 study involving 387 IUI cycles, LBR of 19% and 1.5%, respectively, were achieved when inseminations were carried out with semen of men with normal and abnormal SDF values, measured by the TUNEL assay (6). These results are consistent of those of a recent systematic review and meta-analysis of ten studies and 2839 cycles that showed a strong association between SDF and IUI outcomes (15). In this study, the relative risk of pregnancy failure by IUI was significantly higher in couples whose male partners had high SDF (RR 0.34; 95% CI 0.22-0.52; I^2^=1.2%; P<0.001).

Thus, for our patients with IUI failure, like the case study under discussion, SDF testing could help guide clinical management, albeit the quality of the evidence supporting this recommendation is not very high (21). For such couples, ART, in particular, ICSI, would be a better alternative to overcome SDF-related infertility, possibly due to the technical differences between the two methods of fertilization (6, 22).

However, despite the better results with ART in cases of high SDF, sperm chromatin damage seems to adversely affect both conventional IVF and ICSI pregnancy outcomes. In a 2017 review aggregating the data from 70 studies and over 17,000 ART cycles, the likelihood of pregnancy failure was higher in couples whose male partners had high SDF (IVF studies: OR 1.15, 95% CI 1.05-1.27, p=0.003; ICSI studies: OR 1.12, 95% CI 1.01-1.25, p=0.025). The magnitude of the effect varied with the type of SDF assay (TUNEL: OR 1.85; 95% CI 1.52-2.26, p<0.0001; SCD: OR 1.16, 95% CI 1.02-1.32, p=0.023; Comet: OR 4.15, 95% CI 3.045.68, p<0.0001; SCSA: OR 1.14, 95% CI 1.04-1.25, p=0.004) (14). Notably, the authors showed that the clinical utility of the test concerning pregnancy prediction increased when female infertility factors were excluded (1704 cycles; OR 1.37, 95% CI 1.11-1.68, p=0.003), thus highlighting the importance of SDF to ART outcomes.

Added to the increase in the risk of pregnancy failure, SDF also increases the risk of miscarriage in pregnancies achieved with the use of both conventional IVF and ICSI (16 studies; RR: 2.2; 95% CI: 1.54-3.03; p<0.00001) (14, 23). Therefore, SDF test results might be used not only to prognosticate ART outcomes but also guide clinical management, as it has been suggested that ICSI with testicular sperm in preference over ejaculated sperm could be a valid option to overcome infertility in cases of high SDF (21).

A possible explanation for the better ICSI outcomes with testicular sperm relates to the ∼3fold lower SDF in testicular specimens than ejaculated counterparts (24). The susceptibility of sperm chromatin to oxidative attack, particularly during epididymis transit, is well-established and might explain the low testicular sperm positivity for SDF among infertile men (25).

The above findings seem to translate in better reproductive outcomes when testicular sperm rather than ejaculated sperm are used for ICSI in couples whose male partners have confirmed high SDF in the semen. In a systematic review of four ICSI studies including 507 cycles, the use of testicular sperm for ICSI improved clinical pregnancy rates (OR 2.42, 95% CI 1.57-3.73; I^2^=34%, p<0.0001), decreased miscarriage rates (OR 0.28, 95% CI 0.110.68, I^2^=11%, p=0.005, and increased LBRs (OR 2.58, 95% CI 1.54-4.35, I^2^=0%, p=0.0003) (26). After that, confirmatory evidence concerning the effectiveness of testicular sperm for ICSI has been reported by several independent groups (reviewed by Lopes & Esteves (27)). Thus, despite the still limited evidence and lack of randomized controlled trials, the above data overwhelmingly suggest that the SDF test could guide management by selecting the couples who might benefit from ICSI with testicular sperm.

As far as the health of resulting offspring is concerned, no study has yet compared ICSI with testicular versus ejaculated sperm when both are available. However, the published data concerning the use of testicular sperm from azoospermic men are overall reassuring with regards to the most critical outcomes (28–30). Moreover, data from the group of Cornell, using comprehensive chromosomal evaluation by next-generation sequencing (NGS) analysis, indicate that testicular sperm have not only lower DNA fragmentation but also lower aneuploidy rates than ejaculated sperm (testicular sperm: 1.2%; ejaculated sperm: 8.4%; p=0.02) (31). Lastly, recent data from our group corroborate the safe utilization of testicular sperm. In a 2019 study evaluating 363 couples undergoing ICSI, we looked at the likelihood of a blastocyst being euploid -by NGS- according to the type of sperm used for ICSI (32). We found no differences in the probability of a metaphase oocyte to turn into a euploid blastocyst when ICSI was carried out with the use of testicular or ejaculated sperm taken from men with high SDF, thus suggesting that testicular sperm is as healthy as, if not healthier than, ejaculated sperm.

## DISCUSSION

Using SSFTs in clinical practice, the clinician can, first of all, make a more accurate diagnosis concerning the male factor contributing to infertility. This might help to identify and treat the underlying conditions with the aim of improving sperm function, possibly impacting positively on delivery rates of healthy offspring. Furthermore, better patient counseling can be provided concerning treatment outcomes, and lastly, results of tests could help us guide clinical management towards more personalized and effective ART.

Let us consider the following scenario. An IVF Center performs about 1,000 cycles a year with an overall clinical pregnancy rate (CPR) of about 40%. According to the best available evidence from 14 IVF and ICSI studies involving 2,756 couples, the risk of miscarriage is increased in couples with high SDF subjected to IVF or ICSI with ejaculated sperm (OR 2.7, 95% CI:1.4-5.1, p=0.003) (13). Translating the OR to plain numbers, it means that this hypothetical Clinic could lose about 82 pregnancies in a year as a result of SDF, thus leading to an absolute LBR reduction of about 20%. Naturally, fertility clinics cannot afford such a loss, and therefore, they should care about SDF testing.

Actually, none of us should ignore the factors affecting the health of sperm and the resulting offspring. Although ICSI is an extraordinary achievement, evidence accumulated over the last 25 years indicates that the health of ICSI offspring might be affected, in particular, when the subfertility is of male origin (30). The health issues potentially related to male subfertility and use of ICSI includes congenital malformations, childhood cancer, psychological and neurological development abnormalities, infertility, and cardiometabolic profile impairment (reviewed by Esteves et al. (30)).

We advocate the use of SSFTs, in particular SDF, to identify and treat the underlying conditions associated with abnormal sperm function (20, 33, 34). For instance, let us consider the case of varicocele, whose pathophysiology is linked to OS that is a well-known causative factor for SDF. Data from 21 studies and 1270 infertile men indicate that varicocele repair improves sperm chromatin integrity with an average absolute reduction in SDF values of about 8% (35). In this review, the reduction in SDF after varicocele repair was shown to be translated into a higher chance of achieving both natural and assisted pregnancies; the mechanism seems to be related to the alleviation of OS. Emerging evidence also indicates that other interventions, including lifestyle changes, treatment of genital tract infections, and FSH therapy could help to reduce SDF (20, 34).

**Figure 1:**
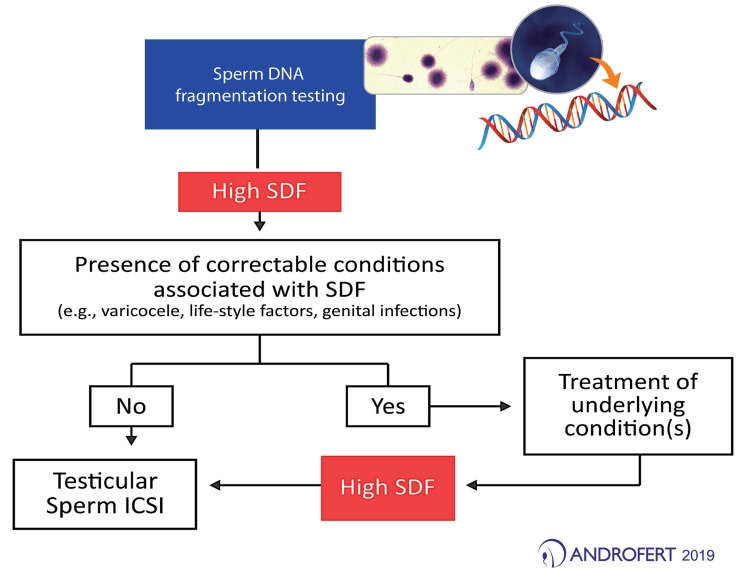
Proposed algorithm for planning intracytoplasmic sperm injection (ICSI) in cases of high sperm DNA fragmentation (SDF).

Therefore, sperm DNA testing can be undoubtedly useful in planning ICSI. A high SDF test result calls for action, which includes the treatment of underlying conditions to improve both sperm chromatin integrity and fertility prospects potentially. When no treatable condition is identified, consideration to ICSI with testicular sperm should be given (Figure-1) (24, 36). However, given the risks associated with sperm retrieval (37–40), ICSI with testicular sperm should be reserved for men with confirmed sperm DNA damage or severe oligozoospermia/cryptozoospermia (41); this is one of the reasons why testing is important.

According to the data of a prospective study by our group comparing reproductive outcomes in 172 oligozoospermic men with high SDF treated by ICSI with ejaculated and testicular sperm, five couples have to be treated by ICSI with testicular sperm (versus ICSI with ejaculated sperm) to achieve one additional delivery in couples whose male partners have oligozoospermia and high SDF in semen (42). Whereas this might represent more invasive treatments for men, it would translate in fewer treatments for women, who are generally the ones carrying the burden.

Lastly, it is easy to criticize the SSFTs based on the grounds of low predictive values and variable thresholds (43, 44). However, it is essential to understand that infertility is a couple's problem; it is, therefore, evident that a single test from just one side will be always limited to provide the full picture (45–50). More important is to acknowledge the fact that high SDF increases the risk of an adverse reproductive outcome, even with ICSI, and that the risk is modulated by female age. The equation relating the continuum of SDF values and maternal age makes much more sense than fighting about absolute thresholds and predictive values, in a scenario where pregnancy rates in the best circumstance will rarely go beyond 50%. Naturally, sperm DNA testing does not replace the adequate male infertility evaluation, but they can certainly add independent information that could help us to offer better care to our patients. So, let us think clinical (and less critical).

## FINAL REMARKS

As recently highlighted in an editorial by Carrel and Hotaling (51), we as individuals and as a medical community providing care to infertility patients, including urologists, gynecologists, and IVF specialists, should ask whether we are providing the best care to our patients and the child yet to be born, by ignoring the health of the sperm. We must also confront the fact that ICSI is overused and the male factor is commonly overlooked. The financial incentives affecting the decision to bypass the male factor infertility through ICSI is not without adverse consequences. The existing data clearly indicate that sperm DNA damage is associated with reproductive health issues in the male and in the embryo. Thus, the use of sperm DNA testing is evidence-based and should be implemented by ART Clinics and doctors not yet using these assays. The primary objectives are to improve IUI and ART success, but more importantly, to improve the health of the father and resulting offspring.
